# IPDmada: An R Shiny tool for analyzing and visualizing individual patient data meta‐analyses of diagnostic test accuracy

**DOI:** 10.1002/jrsm.1444

**Published:** 2020-09-09

**Authors:** Junfeng Wang, Willem R. Keusters, Lingzi Wen, Mariska M. G. Leeflang

**Affiliations:** ^1^ Julius Center for Health Sciences and Primary Care UMC Utrecht, Utrecht University Utrecht The Netherlands; ^2^ Centre for Evidence‐Based Chinese Medicine Beijing University of Chinese Medicine Beijing China; ^3^ Department of Clinical Epidemiology, Biostatistics and Bioinformatics Amsterdam UMC, Amsterdam Public Health, University of Amsterdam Amsterdam The Netherlands

**Keywords:** covariate‐adjusted ROC, diagnostic test accuracy, individual meta‐analysis, Shiny, summary ROC

## Abstract

**Background:**

Individual patient data meta‐analyses (IPD‐MA) are regarded as the gold standard for systematic reviews, which also applies to systematic reviews of diagnostic test accuracy (DTA) studies. An increasing number of DTA systematic reviews with IPD‐MA have been published in recent years, but there is much variation in how these IPD‐MA were performed. A number of existing methods were found, but there is no consensus as to which methods are preferred as the standard methods for statistical analysis in DTA IPD‐MA.

**Objectives:**

To create a web‐based tool which integrates recommended statistical analyses for DTA IPD‐MA, and allows researchers to analyse the data and visualize the results with interactive plots.

**Methods:**

A systematic methodological review was performed to identify statistical analyses and data visualization methods used in DTA IPD‐MA. Methods were evaluated by the authors and recommended analyses were integrated into the IPDmada tool which is freely available online with the user interface developed with R Shiny package.

**Results:**

IPDmada allows users to upload their own data, perform the meta‐analysis with both continuous and dichotomized tests, and incorporate individual level covariate‐adjusted analysis. All tables and figures can be exported as .csv or .pdf files. A hypothetical dataset was used to illustrate the application of IPDmada.

**Conclusions:**

IPDmada will be very helpful to researchers doing DTA IPD‐MA, since it not only facilitates the statistical analysis but also provides a standard framework. The introduction of IPDmada will harmonize the methods used in DTA IPD‐MA and ensure the quality of such analyses.

**Highlights:**

- IPDmada is a newly developed web‐based tool for performing statistical analysis of individual patient data meta‐analysis of diagnostic accuracy and visualizing the results.
- The tool is freely available to all the researchers, and requiring no installation of statistical software/packages.
- The tool has an user‐friendly interface, and allows meta‐analysis on both dichotomized and continuous test results. Researchers can easily use this tool to investigate the threshold effect and covariate effect on the summary accuracy.
- The introduction and implementation of IPDmada will serve as a useful tool for DTA IPD‐MA and increase the quality of such studies.

## 1 BACKGROUND

Compared to conventional meta‐analysis (ie, meta‐analysis of interventions), meta‐analysis of diagnostic test accuracy (DTA) is more prone to heterogeneous studies due to both the patient characteristics and the threshold effect.^1^ Individual patient data meta‐analyses (IPD‐MA) may help to overcome such difficulties. The richness of individual patient data, in both patient‐level variables and the quantity of data, can facilitate advanced analyses which could not be done with aggregated data, but it also leads to big variation in the methods of IPD‐MA.

However, not like other types of meta‐analyses, the standard approach for DTA IPD‐MA is underdeveloped. Many different ways of performing DTA IPD‐MA were used in practice, but there was no clue which are the preferred methods.

In this technical notes, we will introduce our methodological review of statistical methods in DTA IPD‐MA in practice, and give our recommendations on preferred analyses. Based on the findings of the review, we developed a web‐based tool IPDmada with R Shiny, which integrates all the preferred statistical analyses for DTA IPD‐MA. IPDmada allows researchers to analyse the data, investigate the study‐level and/or patient‐level heterogeneity as well as the threshold effect, and finally visualize the results with interactive plots.

## 2 METHODS IN INDIVIDUAL PATIENT DATA META‐ANALYSIS OF DIAGNOSTIC ACCURACY

### 2.1 Review of methods used in practice

A systematic search was performed to identify published articles containing IPD meta‐analyses of DTA studies. Embase and Medline databases were searched from 2000 to 2013 (and updated to 2014) by using a well validated search strategy from a research on DTA meta‐analyses and adjusted for IPD. Twenty‐nine DTA IPD‐MA articles published between 2006 and 2014 were selected as the subjects for the final analysis. The detailed search strategy and a flow chart for study selection were provided in Data S2.

Articles included for the analysis were carefully examined and data was extracted with a data extraction form (in Data S2) designed by two authors (Junfeng Wang and Mariska M. G. Leeflang). Information on statistical methods used was extracted by one author with good knowledge in statistics in test evaluation (Junfeng Wang) and figures and plots for data visualization were extracted by another author (Lingzi Wen).

### 2.2 Recommended analyses

Summary of findings related to statistical analyses are briefly described here, and the full list of findings is provided in Data S2, which was presented in 2015 Cochrane Colloquium.^2^

DTA IPD meta‐analysis is most useful when original continuous test results were provided for each individual. If only binary test results (ie, test result is positive or negative) were provided on individual level, this information could be obtained from the degenerated 2‐by‐2 tables as well, such IPD‐MA will not have much added value compared with meta‐analysis on aggregated data.

In IPD‐MA, researchers not only collected test results of index test(s) and reference standard, but also pursued other patient‐level information, which included but not limited to patient characteristics, for example, sex, age, and BMI, and values of other biomarkers or medical tests from the same individual.

IPD meta‐analysis of continuous test results and patient‐level covariates offers the possibility of performing advanced analyses, such as investigating the effect of threshold, for example, reconciling thresholds from primary studies to a predefined value, or using optimal threshold within each primary study; directly using continuous test results instead of dichotomizing them, which facilitated the calculation of area under ROC curve instead of summary ROC curve in the conventional meta‐analysis; adjusting for baseline differences in study‐level as well as patient‐level characteristics if these characteristics have some confounding effects.^3^

We found that, like DTA meta‐analysis of aggregated data, sensitivity and specificity and area under the ROC curve (AUC) are still the most commonly used measures of test accuracy in DTA IPD‐MA. So we will provide all these performance measures for all primary studies in our tool.

Given individual level data is available, regression models including fixed and random effects, multilevel and GEE logistic regression models are also used in DTA IPD meta‐analysis. However, these models were used for multivariable prediction model development, which is out of the scope of DTA meta‐analysis. IPD‐MA of diagnostic modeling studies was discussed elsewhere.^4^

So in the IPDmada tool, we will focus on advanced statistical analyses facilitated by IPD which could not be done with aggregated data, in particular cut‐off value analysis and covariate adjustment analysis.^5^ In the meanwhile, analyses of aggregated data in the two‐stage approach were also provided by using bivariate model^6^ and HSROC model,^7^ which are the standard approaches and best practices of DTA meta‐analyses.^8^

## 3 THE IPDMADA TOOL

We used R Shiny package^9^ to create a web‐based user interface, which allows interactive data analysis and visualization with R. The advantage of this framework is, we can make use of the R environment and existing R packages, while users do not need any experience with using R.

IPDmada was implemented on the webserver provided by shinyapps.io, so installation of R or RStudio on user's device is not needed. The web‐based tool can be accessed via the following link https://jwang7.shinyapps.io/ipdmada/.

We aimed to design the interface in an user‐friendly way, and also guide the users to conduct the IPD‐MA step by step. Thus, three pages were created for “Import Data,” “Analysis of dichotomized test results,” and “Analysis of continuous test results.” A sidebar is available on the left side of each page, to display the options available for the analyses on the same page, which can be defined by users. Operation of the tool can be simply done by “point and click” on the web browser. All the analysis results will be presented in tables and figures, which can be exported as .csv or .pdf files.

In “Analysis of dichotomized test results,” sensitivities and specificities from primary studies can be redefined by tuning the thresholds, either with a predefined value, an optimal value or any value input by the “slider” bar, and real time forest plot and SROC curve will be plotted accordingly; in Analysis of continuous test results, IPD from primary studies will be integrated into one big dataset and ROC curves for each study and covariate‐adjusted analysis will be plotted and summary AUC will be presented in a forest plot. In Section 4, we provided an illustrative example of how to use this tool.

## 4 AN ILLUSTRATIVE EXAMPLE WITH HYPOTHETICAL DATA

In most of the published IPD‐MA, patient‐level data was not provided. So a hypothetical individual patient data (provided as Data S2) was used as an example for illustrative purpose. There were 15 primary studies evaluating one index test and all studies reported the test results as a continuous variable. Disease status and some patient‐level covariates were also provided. We assumed no missing values in this example dataset. When missing values exist in one variable, they will be removed from the analyses including that variable and kept for other analyses.

### 4.1 Import Data

On the “Import Data” page, users can upload their pooled individual patient data with a comma delimited (.csv) file. The separator can be either comma, semicolon or tab, users are reminded to choose the appropriate one for their data. The dataset should be constructed as follow: three columns contain study name (can be author's name or numerical code, but the variable name must be “Study”), test results of index test (must be a numerical variable named “test.results”), and disease statues (must only contain 0 or 1 and named “disease”). Other columns are for covariates, there are no limitations on the numbers and names of the covariates.

After data was imported, study ID was automatically generated as a sequential number. If display option is Head, then only the first six rows will be presented; option All should be selected to see the full dataset. Beside the raw data, summary statistics of each primary study are also calculated with R package table1 and shown in tab “Summary table.”

**FIGURE 1:**
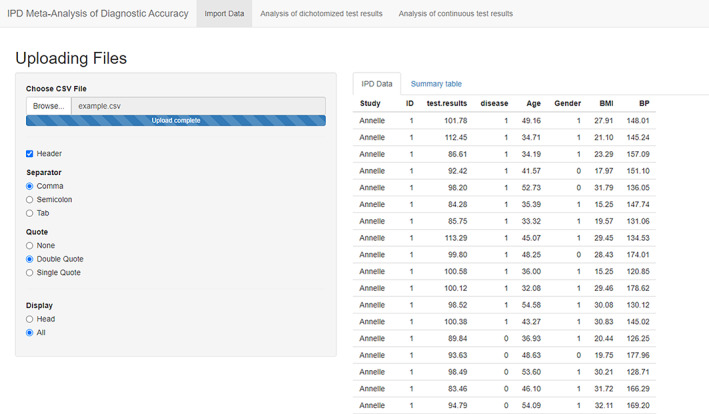
Screenshot of Import Data [Colour figure can be viewed at wileyonlinelibrary.com]

Data entry of the example data is shown in Figure 1.

### 4.2 Analysis of dichotomized test results

The second page is “Analysis of dichotomized test results,” which provides three options to define the positivity threshold of the index test. With the first option “User selected threshold per study,” N (N = number of primary studies included in MA) slider bars were created and user can tune the threshold value for each study. The default value is set to the median of all the test results. If the option “Optimal threshold” was selected, all slider bars will disappear and optimal threshold defined by optimizing Youden's index was calculated for each primary study with R package pROC.^10^ The option “Pre‐defined threshold” will ask the user to enter a number in the box below.

Please note that, using optimal threshold in primary studies may lead to bias estimates of test performance^11^ in meta‐analyses, and using different thresholds may not be clinically meaningful. These analyses were mainly for exploration and sensitivity analysis purposes, and same threshold should be used across all primary studies to get the performance estimates. The properties of this threshold should be known to the clinicians.

**FIGURE 2:**
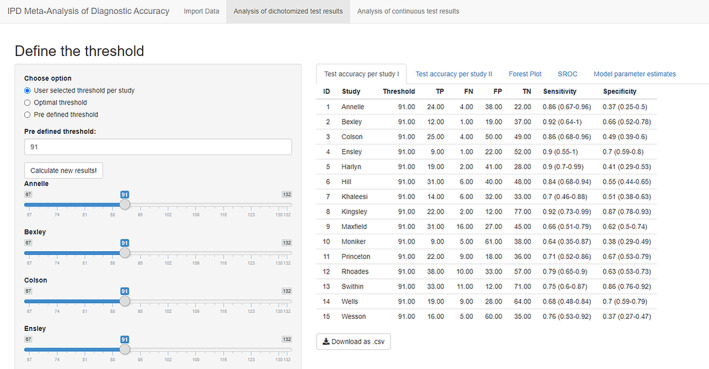
Screenshot of Analysis of dichotomized test results: Test accuracy table [Colour figure can be viewed at wileyonlinelibrary.com]

After the threshold values had been defined as described above, users need to click “Calculate new results!,” then all the results will be presented in the five tabs on the right side. The tab “Test accuracy per study I” shows the threshold value (as define on the left sidebar), true positive number (TP), false negative number (FN), false positive number (FP), true negative number (TN), and sensitivity and specificity according to these numbers (Figure 2). The tab “Test accuracy per study II” shows further analysis results including positive predictive value (PPV), negative prediction value (NPV), likelihood ratio test positive (LR+), likelihood ratio test negative (LR−), diagnostic odds ratio (DOR), and the area under ROC curve (AUC). All these test accuracy measures were calculated with R packages epiR^12^ and pROC.

**FIGURE 3:**
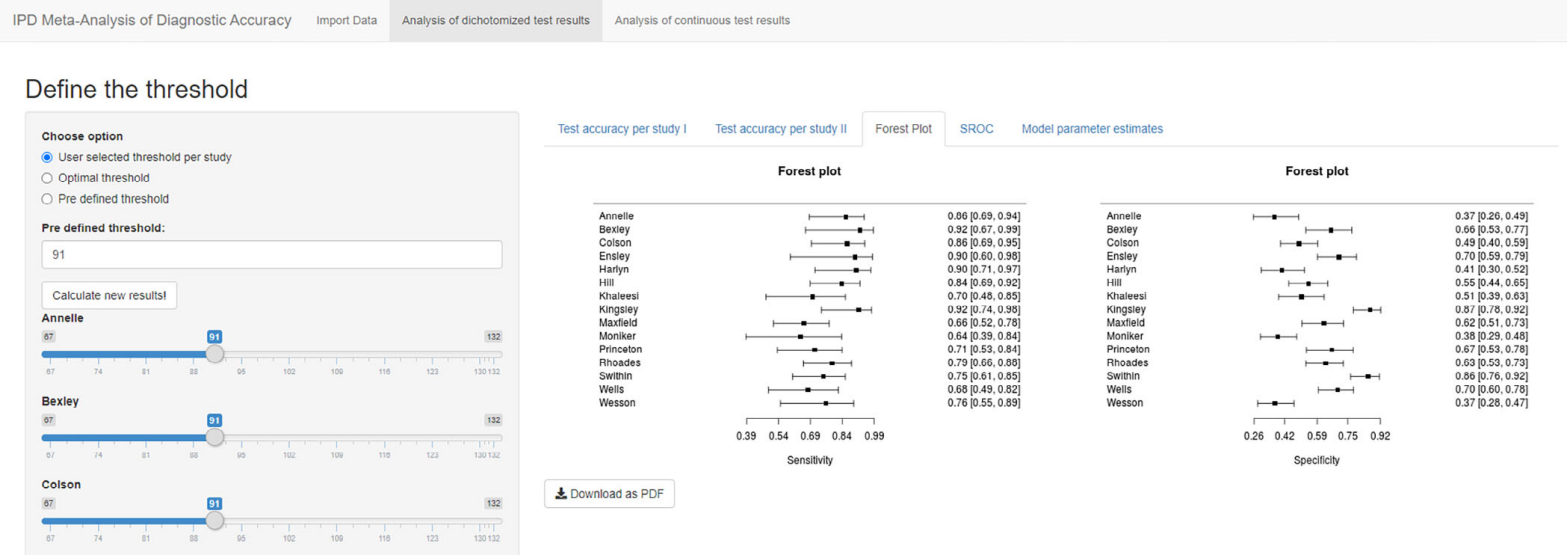
Screenshot of Analysis of dichotomized test results: Forest Plot [Colour figure can be viewed at wileyonlinelibrary.com]

**FIGURE 4:**
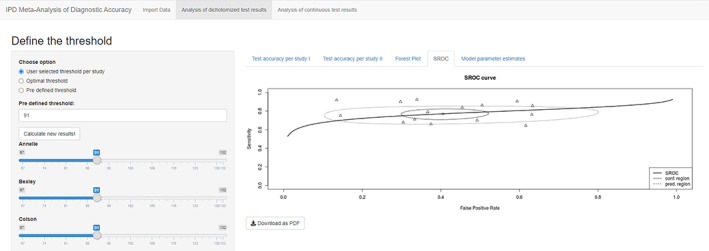
Screenshot of Analysis of dichotomized test results: SROC curve [Colour figure can be viewed at wileyonlinelibrary.com]

The third tab “Forest Plot” presents the forest plots for sensitivity and specificity separately (Figure 3), and the fourth tab “SROC” presents the summary ROC curve derived from bivariate model^6^ of sensitivity and specificity implemented in R package mada^13^ (Figure 4). The model parameter estimates for bivariate model and HSROC model^7^ were provided in the last tab “Model parameter estimates.”

All tables and figures shown in the tabs can be downloaded as .csv files or .pdf files.

### 4.3 Analysis of continuous test results

The second page is “Analysis of continuous test results,” which allows direct analysis of the index test on its continuous scale. All the covariates read from the imported data are presented on the left sidebar with a radio button, and one covariate can be chosen for the covariate‐adjusted analyses.

**FIGURE 5:**
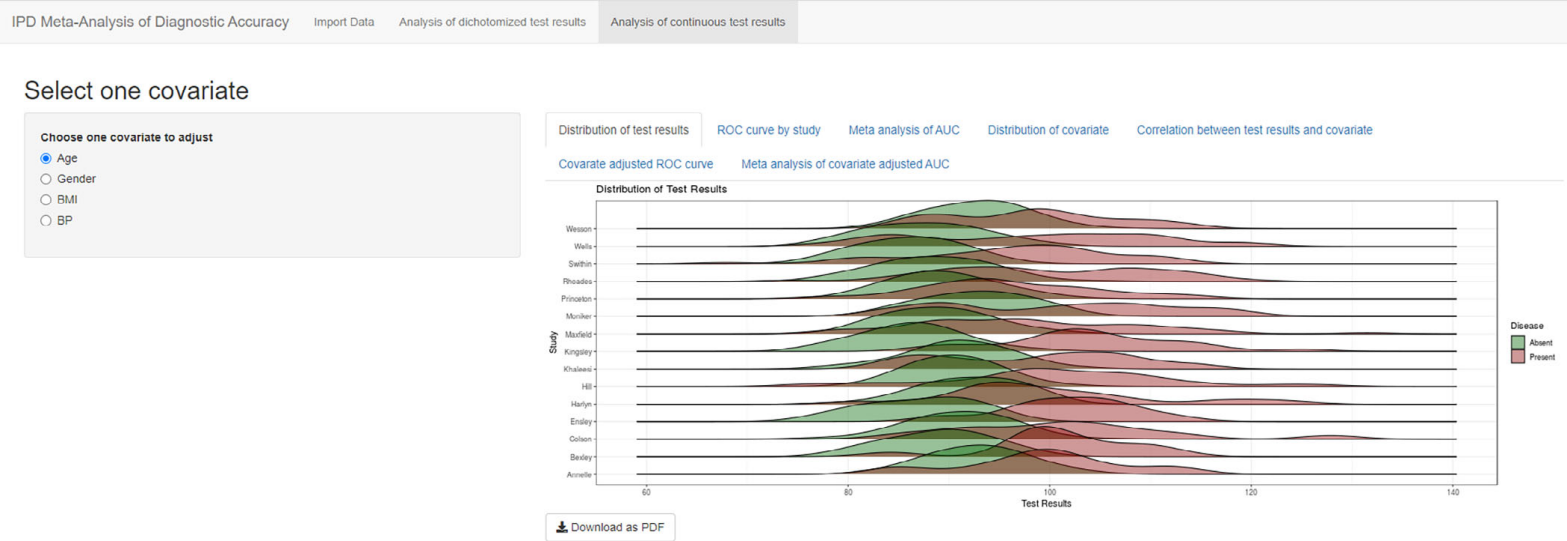
Screenshot of Analysis of continuous test results: Distribution of test results [Colour figure can be viewed at wileyonlinelibrary.com]

The first tab “Distribution of test results” shows the distributions of the index test in diseased and nondiseased groups in each study (Figure 5). Ridgelineplot can make multiple density plots of a numeric variable for several groups in a staggered fashion, which is a very informative way to visualize the different distributions of test results in primary studies. R packages ggplot2^14^ and ggridges^15^ were used to generate the ridgelineplot.

**FIGURE 6:**
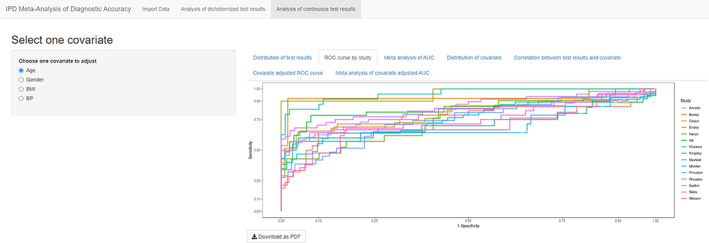
Screenshot of Analysis of continuous test results: ROC curve by study [Colour figure can be viewed at wileyonlinelibrary.com]

**FIGURE 7:**
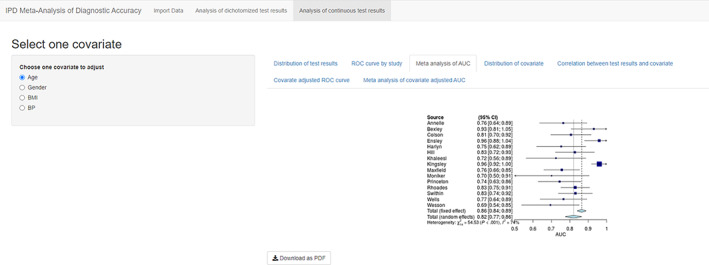
Screenshot of Analysis of continuous test results: Forest Plot [Colour figure can be viewed at wileyonlinelibrary.com]

The second tab “ROC curve by study” shows all ROC curves from primary studies in one plot with R packages ggplot2 and plotROC^16^ (Figure 6). The third tab “Meta‐analysis of AUC” shows both fixed effect and random effect meta‐analyses of AUC^17^ in a forest plot, generated by R meta package^18^ (Figure 7).

**FIGURE 8:**
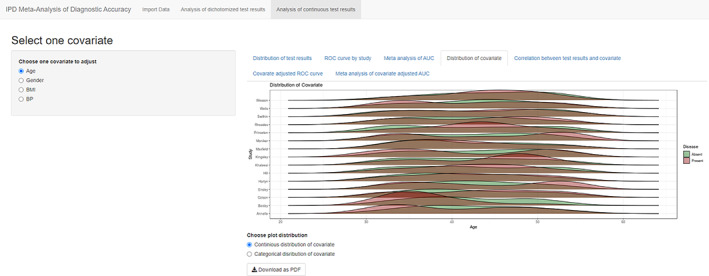
Screenshot of Analysis of continuous test results: Distribution of covariate: Continuous covariate [Colour figure can be viewed at wileyonlinelibrary.com]

**FIGURE 9:**
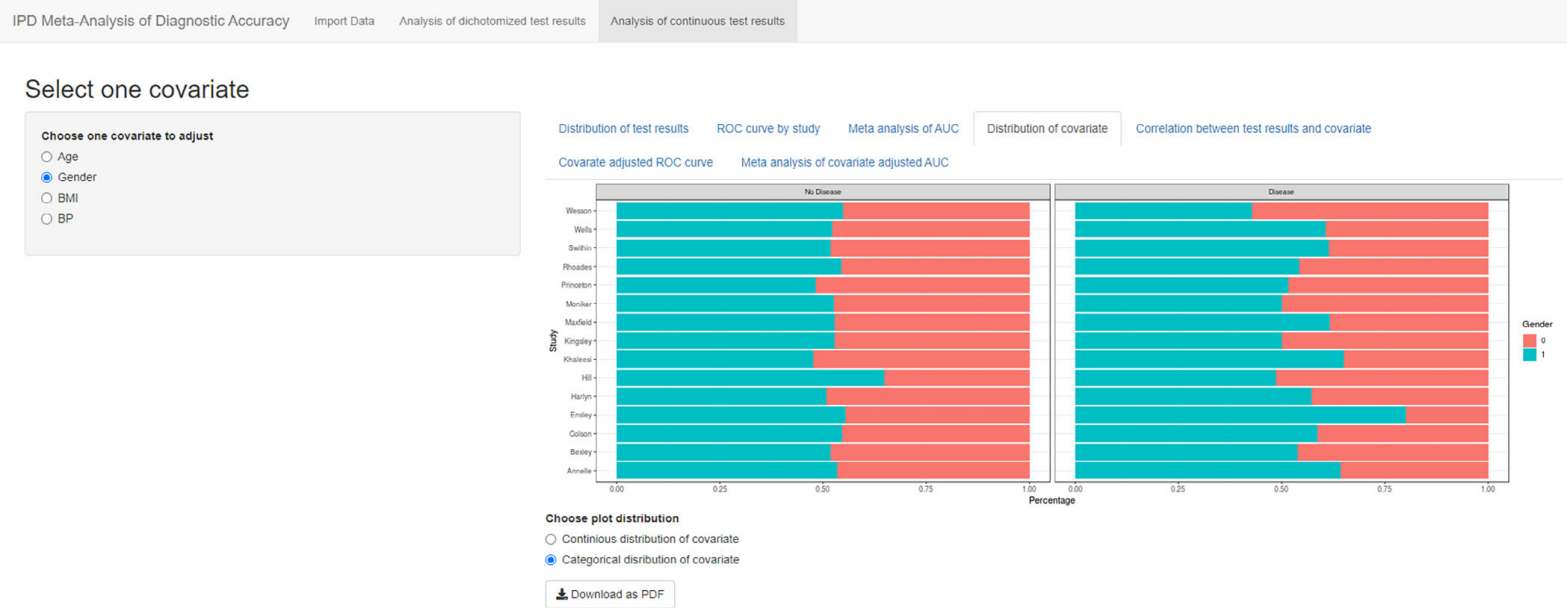
Screenshot of Analysis of continuous test results: Distribution of covariate: Categorical covariate [Colour figure can be viewed at wileyonlinelibrary.com]

**FIGURE 10:**
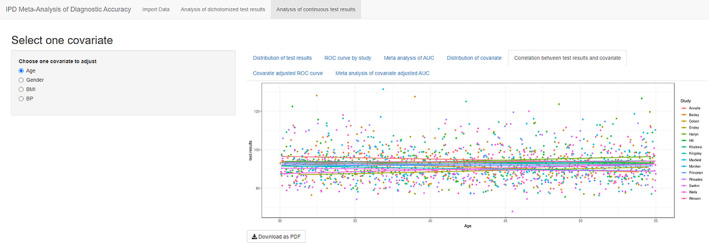
Screenshot of Analysis of continuous test results: Correlation between test results and covariate [Colour figure can be viewed at wileyonlinelibrary.com]

Analyses in table 4 to table 8 are based on the covariate selected from the left sidebar. The fourth tab “Distribution of covariate” is similar to “Distribution of test results” but focused on covariate instead of index test. For continuous covariate ridgelineplot is again used (Figure 8), and for categorical covariate, stacked barchart is used to present the percentages in diseased and nondiseased groups (Figure 9). This figure is aimed to detect different distributions of covariates among primary studies. The fifth tab “Correlation between test results and covariate” presents the relation between test and covariate, by visualizing the study specific liner regression models in a scatter plot (Figure 10).

The last two tabs contain figures similar to Figures 5 and 6, however, the ROC curves and AUC were calculated with covariate‐adjusted ROC analysis^5^ following R package AROC.^19^

## 5 DISCUSSION

In this article, we introduced IPDmada, a newly developed web‐based tool for individual patient data meta‐analysis of diagnostic accuracy.

Web‐based meta‐analysis tools developed with R Shiny gained popularity in recent years. Several tools are available as packages or online tools: MAVIS for meta‐analysis of effect size,^20^ MetaInsight for network meta‐analysis of interventions,^21^ and MetaDTA for meta‐analysis of diagnostic test of study‐level data.^22^ To our knowledge, IPDmada is the first tool available for DTA IPD‐MA.

The development and implementation of IPDmada makes DTA IPD‐MA easier to both researchers with or without strong statistical background. IPDmada will also serve as a useful tool for DTA IPD‐MA and increase the quality of such studies.

Besides the basic analysis provided in IPDmada, the most important functions of IPDmada are interactive analysis of threshold effect and covariate‐adjusted test accuracy, which are the key advantages of using IPD‐MA.

The tool has been back to back tested by the authors with several extreme situations, for example, all the values are missing in one study, wrong input data. It can work properly in such situations.

IPDmada also has some limitations. A comprehensive systematic review was performed to identify the recommended analyses for DTA IPD‐MA, however, the review only included publications till the end of 2014. Future work is needed to consider other new methods introduced recently, and to the best of our knowledge, such methods are still underdeveloped. Furthermore, the selection was based on most often used methods observed in the survey and considered meaningful and useful by the research team, which was a little subjective. It should be seen as a recommendation rather than compulsory requirement, and alternative methods can be still used by researchers in their own IPD‐MAs. Different methods were only compared conceptually, and we did not compare the performance of these methods or check the robustness through simulations, given they are all existing methods and were tested when they were firstly proposed. For data entry, variable names of important variables (study name, test results, disease status) are fixed, users need to prepare their data in the correct format before uploading to the webpage. For covariate analyses, the current version only allows the users to analyze one covariate at one time. Researchers might be interested in adjusting multiple covariates simultaneously in one model, and we are planning to add this function in future updates. In all analyses, complete cases analyses were performed when there were missing values in primary studies. Advanced methods, for example, multiple imputation, would be a better approach to handle missing data. However, multiple imputation may rely on strong assumptions on the mechanisms of missingness. The imputed datasets need to be carefully examined, and analyses of multiply imputed data are also complicated. This requires more statistical knowledge from the user and does not fit the purpose of this easy‐to‐use tool.

IPDmada only has an online version for now, since it is easier for maintenance and update. Users may want to run the analysis locally at their own device, considering IPD usually contains confidential patient‐level data. We are working on the IPDmada package to fulfill this requirement.

It should be noted that, a easy‐to‐use tool is sometimes a double‐edge sword: it provides convenience to researches but also lowers the knowledge requirement for performing the analyses. Even a perfect tool cannot by itself ensure that the data will be analyzed properly and the results will be interpreted correctly. It is highly recommended and encouraged to have a methodologist and a statistician in a review team.

## CONFLICT OF INTEREST

The author reported no conflict of interest.

## AUTHOR CONTRIBUTIONS

Junfeng Wang and Mariska M. G. Leeflang designed the methodology review and recommended methods for DTA IPD‐MA. Junfeng Wang and Lingzi Wen reviewed selected articles and extracted information. Junfeng Wang and Willem R. Keusters designed and developed the Shiny tool. Junfeng Wang, Willem R. Keusters, and Lingzi Wen generated data and tested the tool. All authors provided a substantial contribution to the design and implementation of the web tool, as well as writing or editing the manuscript, and approving the final version.

## Supporting information


**Data S1**. Supporting Information.

**Data S2**. Supporting Information.

## Data Availability

The data that supports the findings of this study are available in the supplementary material of this article.
