# Supplementary material for: IPDmada: An R Shiny tool for analyzing and visualizing individual patient data meta‐analyses of diagnostic test accuracy
Source: Res Synth Methods. 2020 Sep 9;12(1):45–54. doi: 10.1002/jrsm.1444 (PMC7821168; doi:10.1002/jrsm.1444)
Supplement: Supplementary file 1 — Data S1. Supporting Information. [file JRSM-12-45-s001.pdf]

IPDmada: An R-shiny tool for analyzing and visualizing individual patient data meta-analyses of diagnostic test accuracy

Junfeng Wang[1,*,#], Willem R. Keusters[1,*], Lingzi Wen[2], Mariska M.G. Leeflang[3]

1 Julius Center for Health Sciences and Primary Care, UMC Utrecht, Utrecht University, Utrecht, the Netherlands

2 Centre for Evidence-Based Chinese Medicine, Beijing University of Chinese Medicine, Beijing, China

3 Department of Clinical Epidemiology, Biostatistics and Bioinformatics, Amsterdam UMC, Amsterdam Public Health, University of Amsterdam, Amsterdam, the Netherlands

* These authors contributed equally

# Corresponding to: Junfeng Wang, j.wang-4@umcutrecht.nl

Appendix

1. Search strategy for Embase and Medline (via Ovid)

1. systematic.mp. [mp=ti, ab, sh, hw, tn, ot, dm, mf, dv, kw]

2. limit 1 to "reviews (best balance of sensitivity and specificity)"

3. predict*.ti,ab.

4. test.ti,ab.

5. tests.ti,ab.

6. 4 or 5

7. 2 and 3 and 6

8. screen*.mp. [mp=ti, ab, sh, hw, tn, ot, dm, mf, dv, kw]

9. 2 and 8

10. monitoring.mp. [mp=ti, ab, sh, hw, tn, ot, dm, mf, dv, kw]

11. 2 and 10

12. "multiple tests".mp. [mp=ti, ab, sh, hw, tn, ot, dm, mf, dv, kw]

13. 2 and 12

14. "diagnostic test accuracy".mp. [mp=ti, ab, sh, hw, tn, ot, dm, mf, dv, kw]

15. DTA.ti,ab.

16. exp "sensitivity and specificity"/

17. specificit*.tw.

18. "false negative".tw.

19. accuracy.tw.

20. 14 or 15 or 16 or 17 or 18 or 19

21. 2 and 20

22. 7 or 9 or 11 or 13 or 21
23. IPD.ti,ab.
24. individual.ti,ab.
25. 23 or 24
26. 22 and 25
27. limit 26 to (english language and yr="2000 -2013")

2. Identification of DTA IPD meta-analyses

2223 publications were identified by the search performed in the end of 2013 and 381 additional publications were identified by an updated search in the end of 2014. 1585 articles were left for title and abstract screening after duplicated articles were removed. 78 articles were considered for further investigation and full-text were retrieved for these potential inclusions. One additional article was identified by manual search and reference check. In total 79 articles underwent full text check and 50 of them were excluded, so at last 29 DTA IPD meta-analyses articles published between 2006 and 2014 were selected as the subjects for the final analysis (see Figure S 1).

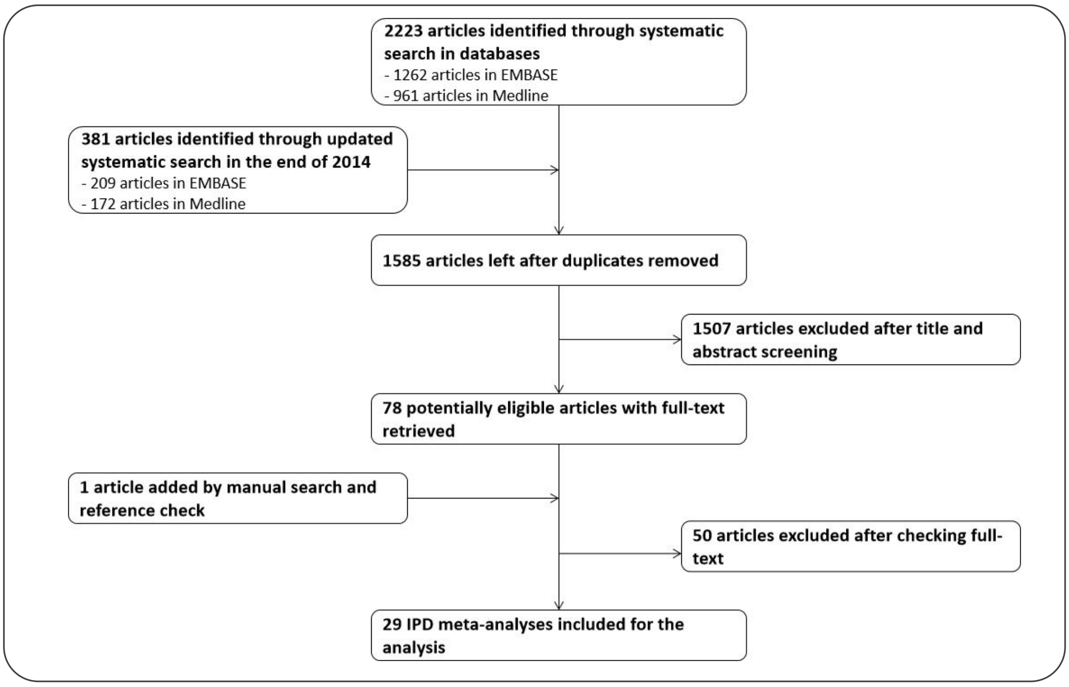

Figure S 1 Flow diagram of study selection process

3. **Data extraction form**

**Type of IPD meta-analysis**	IPD meta-analyses were classified into: 1, Systematic review with IPD meta-analysis; 2, Standalone IPD meta-analysis; 3, Protocol for IPD meta-analysis
**Publication year**	The year when the IPD meta-analysis was published
**Methods for IPD collection**	Whether IPD were obtained from primary study authors or by other methods (specified)
**Number of studies eligible for inclusion**	The total number of studies met the inclusion criteria of the review question
**Number of studies provided IPD**	The number of studies provided IPD to the IPD meta-analysis
**Number of patients eligible for inclusion**	The total number of patients from studies met the inclusion criteria of the review question
**Number of patients included in IPD meta-analysis**	The number of patients finally retrieve and included in the IPD meta-analysis
**Representativeness of IPD**	Whether the authors examine the representativeness of IPD for the whole study population; if the answer is "yes" then the method used will be recorded as well
**Strategies to use IPD**	Strategies were classified into: 1, Analysis with IPD only; 2, Combined analysis with IPD and aggregated data; 3, Separate analyses with IPD and with aggregated data
**Measure of test accuracy**	Open question, any measures of test accuracy reported in the IPD meta-analysis were recorded
**Perspective of test evaluation**	The perspective of a DTA meta-analysis could be estimating the summary accuracy or modelling the probability (prevalence function)
**Statistical methods used**	Open question, any statistical methods used in the IPD meta-analysis were recorded
**Statistical packages**	Open question, any statistical softwares or packages used for analysis
**Additional analysis**	Open question, the kind of additional analysis was recorded
**Missing value**	How missing values were handled in the IPD meta-analysis
**Figures**	All the figures presented in the article
**Comments**	Any other comments related to the IPD meta-analysis

4. Summary results of the DTA IPD meta-analyses survey

Number of DTA IPD meta-analyses published per year

IPD meta-analysis has gained its popularity since the new century. The publication frequency of IPD meta-analysis had a dramatic rise from single figures in the early 1990s to nearly 50 a year since 2005 and increased to around 90 in 2011. The first attempt to apply IPD meta-analysis to evaluate diagnostic tests was published in early 2006, but in that study individual-level data was reconstructed with simulation. The first DTA IPD meta-analysis in real sense came later in 2006 as a part of a comprehensive Health Technology Assessment (HTA) report.

DTA IPD meta-analysis is still in its early stage of development. As shown in Figure S 2, from 2006 to 2014, there were no more than 5 DTA IPD meta-analyses published per year. This may due to the limited number of DTA meta-analysis compared to conventional meta-analysis of treatment. Given the experience we learned from conventional IPD meta-analysis, an increasing in DTA meta-analysis could be expected in the coming decades.

There were 4 protocols for DTA IPD meta-analysis published between 2012 and 2014, but none of these protocols had full report published till this review was conducted.

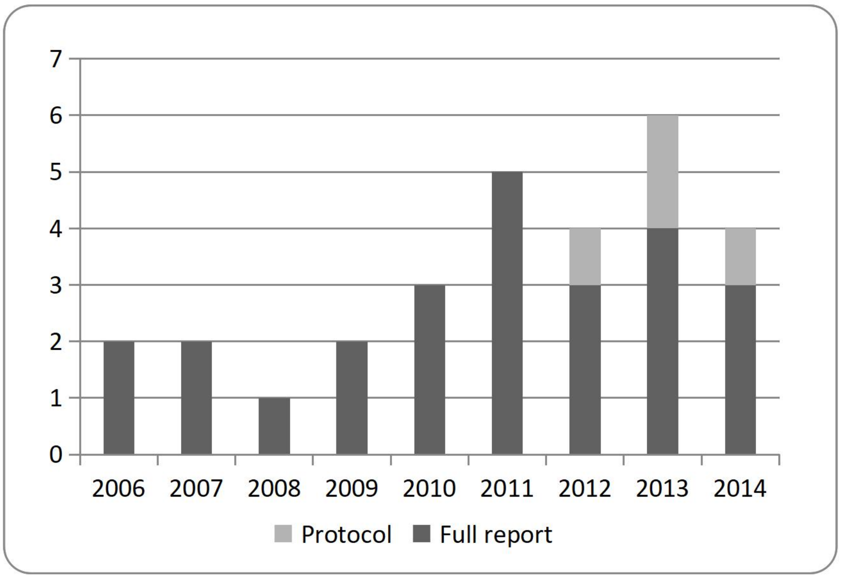

Figure S 2 Number of DTA IPD meta-analyses (protocols) published up to December 2014, identified by a systematic search of Embase and Medline

Types of DTA IPD meta-analyses

Unlike conventional meta-analysis of aggregated data, which is usually used for evidence synthesis in a systematic review, an IPD meta-analysis can be either as a part of a systematic review or a standalone research.

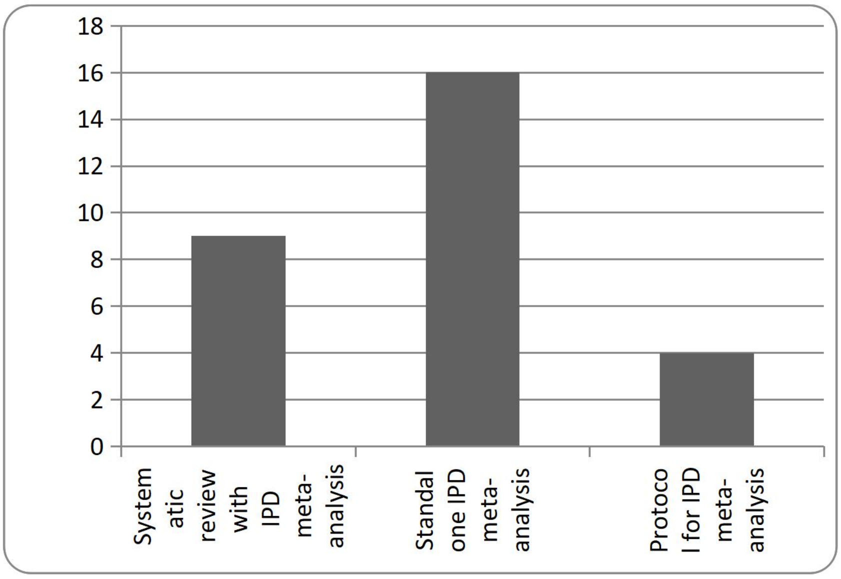

Figure S 3 Different types of DTA IPD meta-analyses

Data collection

Process of obtaining IPD

More than 80% (24/29) of the DTA IPD meta-analyses were performed based on datasets containing individual patient information provided by authors of primary studies.

Simulation was once used to reconstruct individual-level data for each primary study. In this approach, patient characteristics were collected on study level by their mean and SD, thus no more information in addition to the data used for conventional meta-analysis was needed. Although simulation could significantly reduce the efforts of getting access to IPD from primary studies, this practice was not repeated by following researches. The main reason is the limited added value of this kind of IPD meta-analyses to conventional meta-analyses.

In two of the IPD meta-analyses, IPD were extracted from published studies that plotted individual data in the figures with the help of computer software.

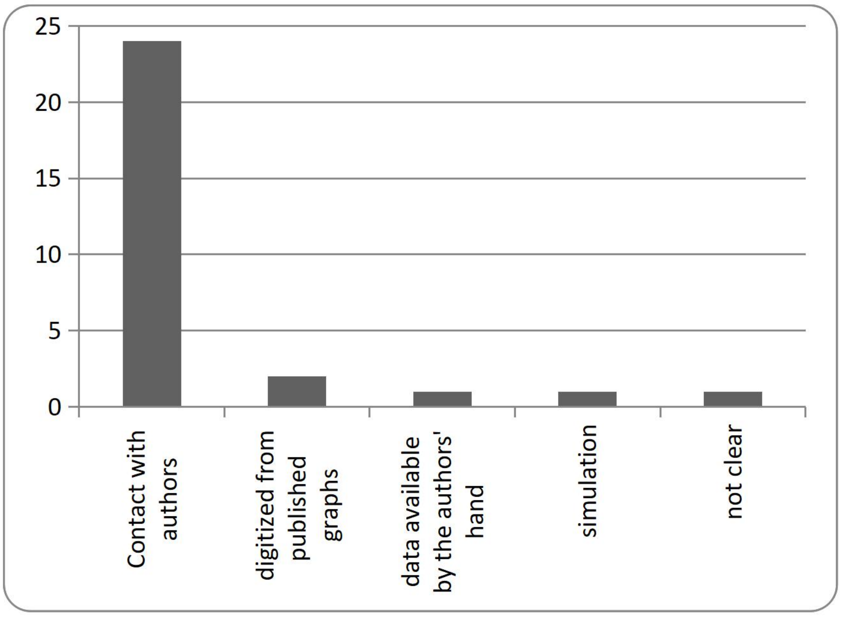

Figure S 4 How individual patient data approached

Although contacting with authors is the common practice of getting data for IPD meta-analysis, there are different directions in data collection when IPD approach initiated: top-down and bottom-up.

When starting with a predefined review question, data collection is initiated with a systematic search to identify all relevant published studies, and then primary study authors are contacted to provide IPD. We can think this as a top-down approach or a systematic review approach. IPD meta-analysis may also be initiated by collaboration: a research group including several authors who agree to share their data to perform an IPD meta-analysis. To the contrary of the former clinical question driven approach, this collaboration based approach is in a bottom-up way.

Number of studies and patients

Figure S 5 shows the number of studies from which IPD were obtained in each of the meta-analyses in the 25 full study reports. 60% (15/25) of the IPD meta-analyses included data from less than 10 primary studies.   Only one of the IPD meta-analyses obtained more than 50 studies.

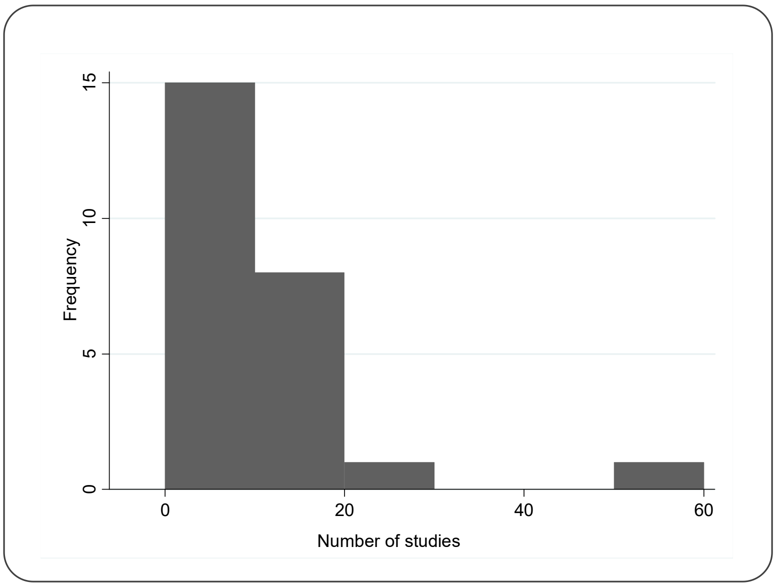

Figure S 5 Number of primary studies provided IPD

Figure S 6 shows the percentage of all eligible studies provided IPD in 23 IPD meta-analyses (two IPD meta-analyses didn't report the number of eligible studies). In the 4 IPD meta-analyses which successfully obtained 100% of primary data, two of them either used simulation method or digitized IPD from published graphs. Except these two IPD meta-analyses, over 60% (13/21) of the DTA IPD meta-analyses only retrieved less than 50% of the eligible primary studies.

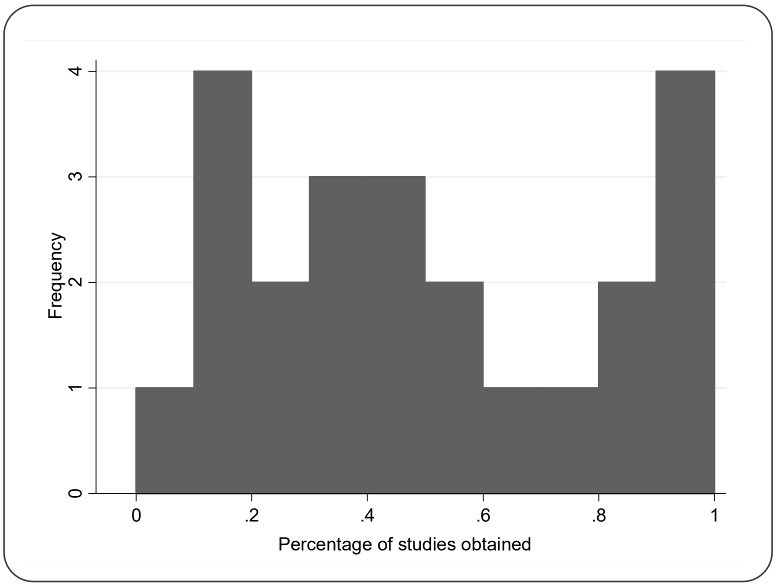

Figure S 6 Percentage of the total eligible studies that provided IPD

60% (15/25) of the IPD meta-analyses had fewer than 2000 patients and only 1 meta-analysis included more than 10000 patients.

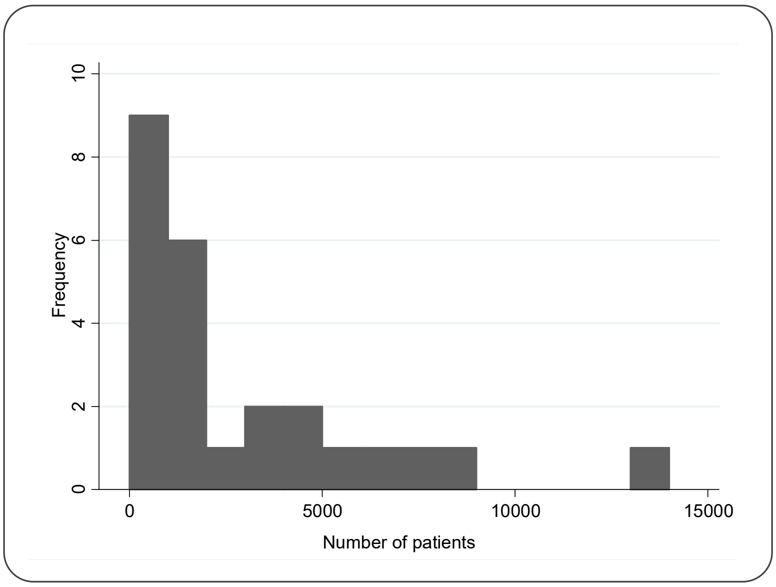

Figure S 7 Number of patients obtained in each DTA IPD meta-analysis

Missing data

Compared to conventional DTA meta-analysis, DTA IPD meta-analysis is more prone to missing data problem. DTA IPD meta-analysis not only collected test results of index test(s) and reference standard (in many cases this information could be got from the degenerated 2-by-2 tables as well), but also pursued other patient level information, which included but not limited to patient characteristics, e.g. sex, age and BMI, and values of other biomarkers or medical tests from the same individual. Since most of the IPD meta-analyses are performed retrospectively, different variables of interest were collected in primary studies, which will lead to huge number of missing values in the combined IPD datasets.

Figure S 8 illustrates how missing values were handled in DTA IPD meta-analyses. Over 70% (21/29) of DTA IPD meta-analyses didn't explicitly mention how to deal with the data missingness in their researches. Four meta-analyses simply excluded the missing values from their analyses. Only four meta-analyses implemented or will implement (protocol) multiple imputation method to the missing variables. The three accomplished DTA meta-analyses using multiple imputation were from the same study group.

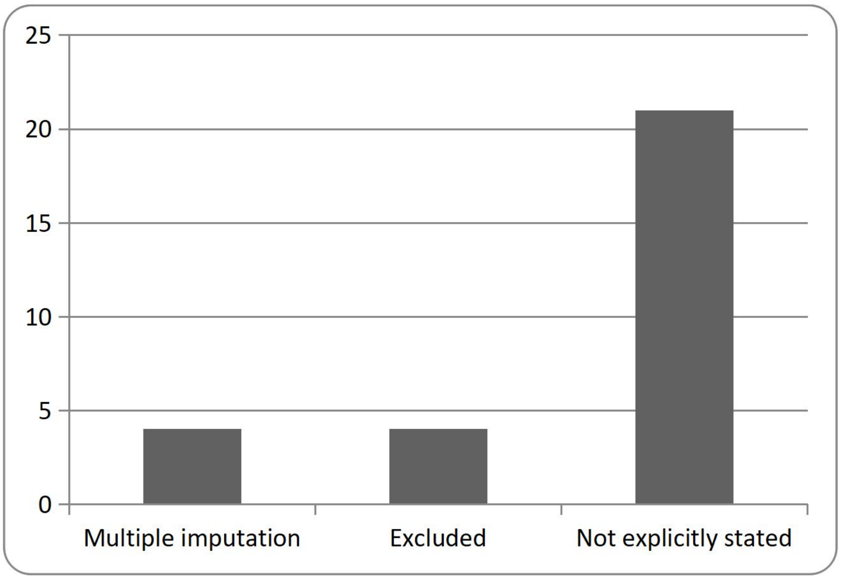

Figure S 8 How missing values were handled in DTA IPD meta-analyses

Representativeness

For those IPD meta-analyses which failed to obtain IPD from all desired primary studies, selection bias (or availability bias) may exist in the summary estimates of test accuracy. Given the large proportion of studies did not provide IPD, the validity of the results from IPD meta-analyses is questionable.

None of the 25 published IPD meta-analysis reports examined the representativeness of their IPD for the whole study population. Only a recently published protocol for IPD meta-analysis tended to perform a sensitivity analysis concerning on the consistency of the results from IPD meta-analysis and non-IPD meta-analysis.

Representativeness can be examined by comparing baseline patient characteristics in cases and controls in studies included in IPD meta-analysis and non-IPD studies or by a sensitivity analysis comparing summary estimates generated from IPD meta-analysis and that from conventional meta-analysis based on studies not included.

Statistical Methods

Measures of test accuracy

Similar with DTA meta-analysis of aggregated data, sensitivity and specificity are most commonly used measures of test accuracy. Over three quarters (21/29) of the DTA IPD meta-analyses calculated summary sensitivity and specificity.

The test results on patient level, which were usually recorded as continuous variables in the original datasets, facilitated the calculation of area under ROC curve instead of summary ROC curve in the conventional meta-analysis.

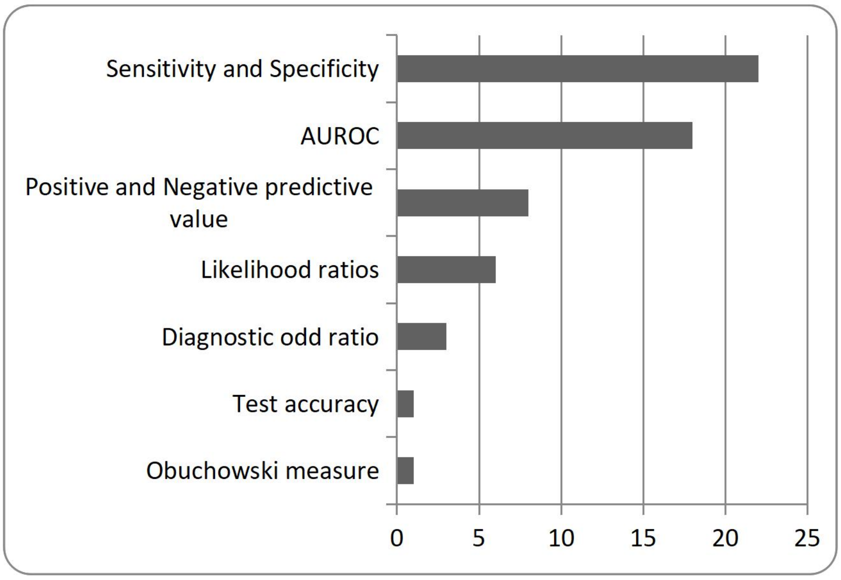

Figure S 9 Measures of test accuracy used in IPD meta-analyses and protocols

Strategies to use IPD

As shown in Figure S 10, in the process of retrieving IPD from primary studies, IPD may not be available from all of them. For these studies, aggregated data on study level are usually available. The authors of IPD meta-analysis may have different choices to use IPD: (1) only use IPD data to perform a pure IPD meta-analysis; (2) perform a conventional meta-analysis with all study level data and an IPD meta-analysis with available IPD; (3) perform an IPD meta-analysis with available IPD and a separate conventional meta-analysis for the rest studies which didn't provide IPD. The difference between the second and third strategies to use IPD is whether studies provided IPD were still included in the conventional meta-analysis.

The authors of over 75% (22/29) of the IPD meta-analyses decided to use only IPD in their analyses. Although there is methodology development of DTA meta-analysis using IPD combined with aggregate data, using only IPD in the analysis is more flexible and easy to implement.

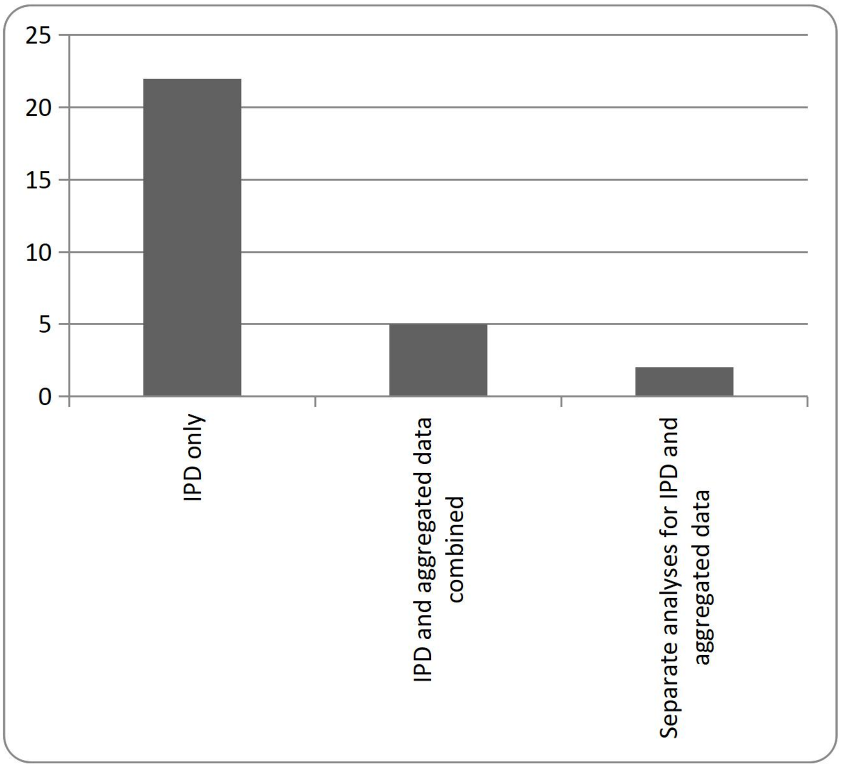

Figure S 10 How IPD was used in meta-analysis

Statistical methods used in IPD meta-analysis

Regression models including fixed and random effects, multilevel and GEE logistic regression models are most commonly used statistical methods in DTA IPD meta-analysis. ROC analyses, especially covariate-adjusted ROC curves were also used independently or together with regression models to estimate summary test accuracy.

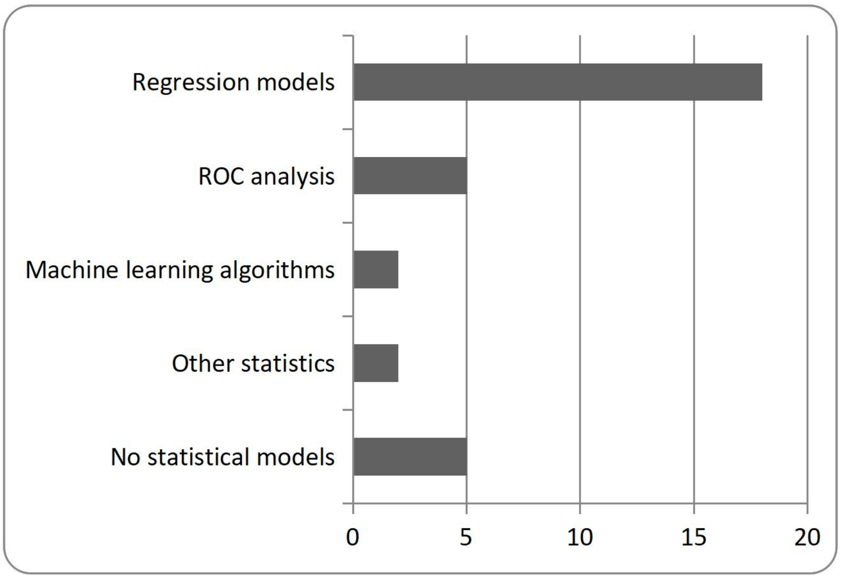

Figure S 11 Statistical methods used in DTA IPD meta-analysis

Additional analyses

The greatest advantage of IPD meta-analysis is that including variables on patient level could facilitate advanced analyses which could not be done with aggregated data. These additional analyses included subgroup analysis, covariate analysis and cut-off value analysis, etc.

In subgroup analysis, patients were regrouped by their characteristics, e.g. age, treatment received, and group specific test performance (sensitivity, specificity or AUROC) were calculated within each subgroup.

In cut-off value analysis, the authors were interested in the optimal cut-off point or evaluate the test accuracy when applying different cut-off points.

Covariate analyses were performed either by including patient level covariates into the regression model or by estimating covariate-adjusted ROC.

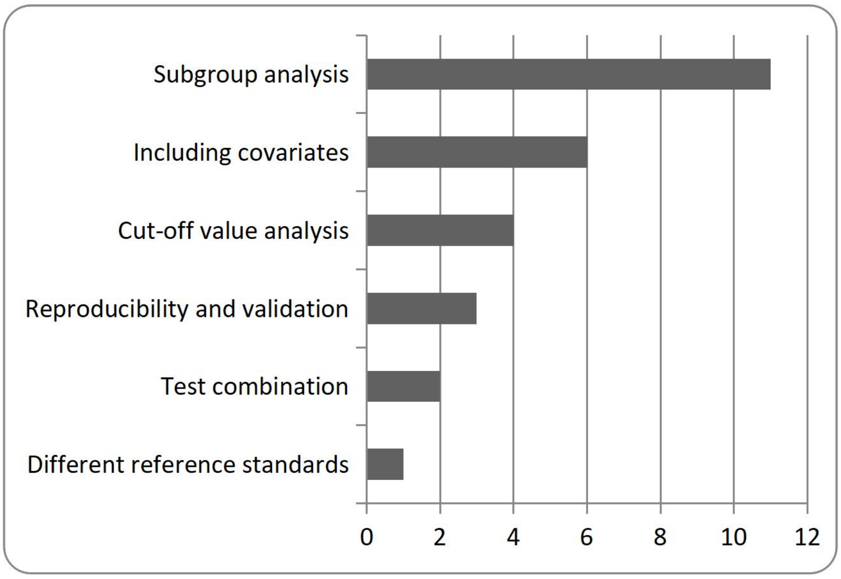

Figure S 12 Additional analysis performed in DTA IPD meta-analysis
